# John Hancocke Wathen

**Published:** 1906-12

**Authors:** 


### Obituary Notices.

---

### JOHN HANCOCKE WATHEN, M.R.C.S., L.R.C.P.

We have to record the death, at the age of 61, of Mr. John Hancocke Wathen, one of the regular attendants at the meetings of the Bristol Medico-Chirurgical Society, and a man well known in professional and political circles in Bristol. His death took place at his residence, Sea Walls, Stoke Bishop, on October 5th, and was somewhat sudden. He had been in failing health for some two or three years, and took but little exercise; he had been conscious that he had a dilatation of the aorta, proved by an X-ray examination two years ago, and the immediate cause of his death was the bursting of this into the œsophagus.

Born at Marloes Court, Pembrokeshire, he received his early education at Fishguard, and afterwards was at school at Weston near Bath. Later on he studied at University College Hospital and the Royal Orthopædic Hospital, and became a Member of the Royal College of Surgeons in 1865. Two years later he took the degree of L.R.C.P. Edinburgh. His first connection with Bristol was his election as Assistant House Surgeon at the General Hospital, and he was afterwards House Surgeon at the West London Hospital.

In 1867 his father, who was one of the ornaments of the profession, an able country doctor, broke his leg, and the son was recalled to Fishguard and practised for thirteen years with eminent success. Here he performed many operations of general surgery and midwifery, and father and son enjoyed the confidence and friendship of the whole countryside. With the object of rising in the profession and having a larger scope for his powers, he came to Clifton in 1880, and started in private practice, hoping to become attached to one of the public medical institutions. He entered into partnership with Dr. Steele, was partner with him for twelve years, and during this period was Medical Officer to the Clifton Wood Industrial School, when he especially turned his attention to skin diseases, and established a skin dispensary of his own in Park Row.

In 1893 he went into the Town Council, and from that date till two years before his death took a prominent part in municipal affairs as a staunch Conservative. He was a member of the Asylum Visiting Committee and the Sanitary Authority, and was created an alderman in 1897—the only other medical man that we can recollect who achieved the distinction of the aldermanic chair being Mr. Thomas Green, Surgeon to the Infirmary, 1844-1864.

Mr. Wathen was a medical officer in the 1st Gloster Artillery Volunteers, and retired after fifteen years' service with the rank of Surgeon-Major. He was President of the Grateful Society in 1895, and in the same year took an active part in the meeting of the Medical Association in this city. He married a daughter of the late Sir George Edwards.

A correspondent writes:—

"For many years I saw a great deal of Mr. Hancocke Wathen, professionally and otherwise, and though I have little to add to the excellent obituary notice printed above, I should like to write a few words about my old friend. Though we had divergent ideas on many subjects, and frequently discussed the dangerous topic politics, both municipal and imperial, and though Mr. Wathen was a Tory of Tories, and often expressed his convictions in terms that left no doubt as to his views, we never fell out, for he always admitted that his opponent could honestly hold the opposite view to his. He was a Protectionist before many who have taken a more prominent position in the near past in advocating Tariff Reform, but was always ready to listen to—and correct—the views held on the other side. In topics more especially connected with our profession he, as might be expected, had his own views, though perhaps it may be said he did not quite appreciate or assimilate the advances recently made in medicine and surgery. Many of us could not help smiling at his frequent references to his past experiences at Fishguard, where no doubt he gained great experience and did much good work. In one particular he had the advantage over many of us who have not served for many years in a country practice, where dispensing chemists are not known—he had a valuable knowledge of the dispensing of drugs, and many little tips for the elegant preparation of medicines. Further, he had the resource in emergencies that comes from the necessary self-reliance gained in a remote country district.

"Years ago few men more enjoyed seeing his friends at his house than Mr. Wathen. He never entertained largely or on a lavish scale, but he was frequently the host of a small dinner party, and his cheerful and kindly disposition made everyone comfortable and happy.

"It was with genuine regret that many of us saw the gradual decline in his health which separated us from a kindly and good-natured friend."

